# *Micromachines*: 5000th Publications Milestone

**DOI:** 10.3390/mi12121573

**Published:** 2021-12-17

**Authors:** Nam-Trung Nguyen

**Affiliations:** Queensland Micro- and Nanotechnology Centre, Griffith University, 170 Kessels Road, Nathan, QLD 4111, Australia; nam-trung.nguyen@griffith.edu.au

*Micromachines* has achieved a major milestone this year. We are pleased to announce that we have passed the 5000th publications milestone. This is a remarkable performance for a journal that just celebrated its 10th anniversary last year. With only 9 papers published in 2010, the journal now publishes over 1300 papers per year. One may think that the large number of papers will dilute the quality of the journal. However, annual citation count and full-text view count have grown exponentially. The impact factor of the journal has been steadily growing year by year and is now approaching a score of three.

In the 11 years of its existence, the journal has evolved from a relative narrow scope to a respected interdisciplinary journal with a broad scope but focused on micro- and nanoscale technologies. Papers published in our journal cover topics ranging from fundamentals and physics to biology and biomedicine, to chemistry, to materials and processing, to engineering and technology. The interdisciplinary nature of the journal is well reflected by its top 10 most cited papers [1,2,3,4,5,6,7,8,9,10]. Many are reviews of microfluidic technologies key for the advancement of chemistry and life sciences such as micro flow sensors [1], microvalves, micropumps [2], micromixers [4,5], cell separation [9] and cell lysis [10]. Fabrication methods such as micro laser machining [3] and polymeric micromachining [7] are also well read and cited. Energy harvesting, particularly for the emerging wearable technology platforms, is also a hot topic. During the COVID-19 pandemic, *Micromachines* is one of the first journals to respond and publish papers related to the efficient management of the disease [8]. The timely and high-quality contributions from all over the world are the key factors for the success of the journal.

I would like to take this opportunity to thank all the authors who have published their works with *Micromachines*. Our editors and reviewers who are the leading experts in their field play a significant role in maintaining the high quality and the fast review process for papers submitted to *Micromachines*, the multidisciplinary board of our editors and reviewers ensures a fair and balanced review process, which is complemented by the exceptionally hard and efficient work of the MDPI editorial team.

This milestone is also dedicated to remembrance of Professor Miko Elwenspoek, who passed away on the 13 April 2021. Professor Elwenspoek was the founding editor of *Micromachines* and was my mentor during a research attachment at the University of Twente back in 1996. On behalf of the editorial board, I offer our condolences to his family and friends. 

I look forward to the next milestones for the journal with our impact factor crossing above three, moving more areas covered by the journal into the first quartile. I am confident that the research community will continue to support *Micromachines* by submitting high-quality papers for publication. Increasing institutional support and mandate from funding agencies will make open access model of journal a success in the near future.

## References

[1] Jonathan T., Kuo W., Yu L., Meng E. (2012). Micromachined Thermal Flow Sensors—A Review. Micromachines.

[2] Au A.K., Lai H., Utela B.R., Folch A. (2011). Microvalves and Micropumps for BioMEMS. Micromachines.

[3] Ahmmed K.M.T., Grambow C., Kietzig A.-M. (2014). Fabrication of Micro/Nano Structures on Metals by Femtosecond Laser Micromachining. Micromachines.

[4] Suh Y.K., Kang S. (2010). A Review on Mixing in Microfluidics. Micromachines.

[5] Cai G., Xue L., Zhang H., Lin J. (2017). A Review on Micromixers. Micromachines.

[6] Guyomar D., Lallart M. (2011). Recent Progress in Piezoelectric Conversion and Energy Harvesting Using Nonlinear Electronic Interfaces and Issues in Small Scale Implementation. Micromachines.

[7] Tsao C.-W. (2016). Polymer Microfluidics: Simple, Low-Cost Fabrication Process Bridging Academic Lab Research to Commercialized Production. Micromachines.

[8] Nguyen T., Bang D.D., Wolff A. (2020). 2019 Novel Coronavirus Disease (COVID-19): Paving the Road for Rapid Detection and Point-of-Care Diagnostics. Micromachines.

[9] Hou H.W., Bhagat A.A.S., Lee W.C.J., Huang S., Han J., Lim C.T. (2011). Microfluidic Devices for Blood Fractionation. Micromachines.

[10] Shehadul Islam M., Aryasomayajula A., Selvaganapathy P.R. (2017). A Review on Macroscale and Microscale Cell Lysis Methods. Micromachines.

